# Hydrogel-based thermoelectrochemical cells for waste heat recovery under passive cooling conditions†

**DOI:** 10.1039/d5mh00771b

**Published:** 2025-07-02

**Authors:** Matteo Bevione, Gopal Narmada Naidu, Giulia Tagliabue

**Affiliations:** a Laboratory of Nanoscience for Energy Technologies (LNET), Faculty of Engineering (STI), École Polytechnique Fédérale de Lausanne (EPFL) Lausanne Switzerland giulia.tagliabue@epfl.ch

## Abstract

With global energy demands rising and the need to reduce greenhouse gas emissions, capturing low-temperature waste heat, which represents ≈ 60% of overall energy waste, offers a compelling pathway to sustainability. Thermoelectrochemical cells (TECs) are promising for converting low-grade heat into electricity but face limitations with liquid electrolytes, including inefficiency and instability under passive cooling. In this work, we introduce hydrogel-based TECs (HyTECs) as a solution to these challenges, leveraging their low thermal conductivity and permeability to sustain larger thermal gradients and stable operation across diverse conditions. We demonstrate that HyTECs achieve a power output of up to 3.5 μW cm^−2^ under passive cooling with a hot temperature of 55–65 °C, comparable to those of state-of-the-art TECs under an externally applied thermal gradient of 10 K cm^−1^. Through thorough experiments and multiphysics modeling, we attribute this performance to the hydrogel's ability to support stable convective cells that enhance redox species transport at the electrode interface. Systematic optimization of key parameters, including redox-pair concentration, electrode separation, and supporting electrolyte levels, revealed that a design with 20 mm electrode spacing, 0.4 M ferro-/ferricyanide, and 0.5 M KCl achieves a power output of 35 mW m^−2^, a Seebeck coefficient of 3.5 mV K^−1^, and a normalized power of 0.6 mW m^−2^ K^−2^. Furthermore, HyTECs exhibit robust performance across orientations (0°–150°) around hot pipes, with a 135° inclination delivering peak power due to enhanced convection and thermal gradients. This work establishes HyTECs as a viable platform for efficient waste heat recovery, providing a foundation for their deployment in real-world energy applications.

## Abstract 2

Hydrogel-based thermoelectrochemical cells sustain large temperature gradients under passive cooling, producing up to 35 mW m^−2^ and a 3.5 mV K^−1^ Seebeck response. They are robust across orientations, enabling scalable waste-heat recovery.

## Graphical abstract

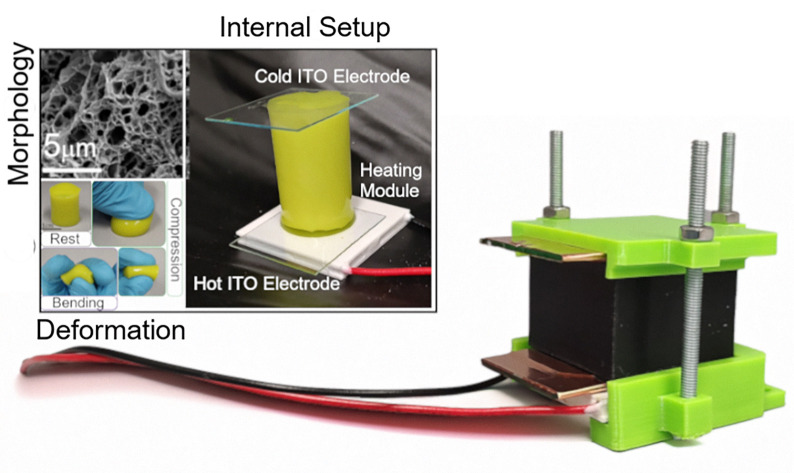

**New concepts**

We report hydrogel-based thermoelectrochemical cells (HyTECs) that operate under passive cooling to convert low-grade waste heat (*T* < 100 °C) into electricity, achieving power outputs of up to 35 mW m^−2^ and Seebeck coefficients of 3.5 mV K^−1^ without externally imposed temperature gradients. In contrast to traditional liquid-electrolyte TECs, these devices use a polyvinyl alcohol hydrogel matrix to minimize leakage, stabilize thermal gradients, and enable controlled convective ion transport without active cooling. We analyse the advantages of HyTECs by comparing them to their liquid counterpart, explaining how the electrode spacing is relevant to guarantee a stable convective cell formation, and how this latter affects the device performance. Through a systematic variation of the redox-electrolyte composition and electrode spacing (optimized at 20 mm), we quantify the relative contributions of thermogalvanic and thermodiffusive effects. This work offers new insight into the role of porous-media transport in thermoelectrochemical energy conversion and suggests design guidelines for integrating solid-like electrolytes into practical waste heat recovery systems.

## 1 Introduction

With rising global energy demands and the urgency to cut greenhouse gas emissions, capturing low-grade waste heat, accounting ≈ 60% of the energy produced,^1^ offers a pivotal opportunity to boost efficiency and sustainability. Advanced technologies to harness this untapped thermal energy are essential for reducing industrial energy footprints.^2^ Several technologies have been developed to address this challenge, such as organic Rankine cycles (ORCs) and absorption refrigeration systems.^3,4^ However, these systems often rely on complex mechanical components, making them costly and difficult to implement for small-scale applications. Nanofluids, such as ferrofluids, have been explored as potential alternatives due to their thermoelectric^5^ or pyroelectric^6^ properties. The photothermal effect in the plasmonic nanofluid, which utilizes plasmonic nanoparticles, has also shown promise for energy applications.^7^ However, issues such as their liquid nature, low output, challenges in encapsulation and manipulation have restricted their use.^8^ Recently, thermoelectrochemical cells (TECs) have gained attention in the scientific community as promising devices capable of directly converting low-grade heat into electrical energy *via* redox reactions.^9–11^

In conventional thermoelectric devices, power generation arises from the Seebeck effect. In contrast, TECs operate *via* a different mechanism, where power generation is primarily governed by redox reactions at the electrode–electrolyte interfaces, driven by a temperature gradient across the cell. This thermal gradient not only induces the required interfacial potential differences but also sustains ionic transport through thermally driven convection and diffusion. This continuous ion migration enables the system to maintain stable operation over extended periods. As a result of this distinct mechanism, TECs can exhibit thermopower values (the TEC counterparts of the Seebeck coefficient) that are two to three orders of magnitude higher than those in conventional thermoelectric systems, approaching ≈ 1% relative Carnot efficiency (see Section S2, ESI†).^12^ Furthermore, the simplicity of TEC design, combined with scalability, the absence of moving parts, adaptability, and potential for miniaturization, makes them highly attractive for waste heat recovery.

Despite these promises, current TEC technologies face significant challenges. One major limitation is their reliance on liquid electrolytes,^13^ which are prone to leakage, evaporation, and drying out during prolonged operation. Furthermore, establishing a stable thermal gradient under passive cooling conditions, *i.e.* where only hot temperature is applied, is critical for realistic waste heat recovery scenarios. However, this remains challenging in liquid electrolytes due to their highly effective heat transport.^14–16^ This leads to low temperature gradients and the formation of incomplete or unstable convective cells, limiting the overall system efficiency. Overcoming these challenges is essential for the practical application of TECs.

Hydrogels have emerged as promising candidates in TEC design due to their quasi-solid structure and low thermal transport.^17^ Composed of a polymer network that can retain a large amount of liquid, hydrogels provide an ionically conductive medium while offering mechanical stability. Their solid-like structure mitigates the risk of leakage and helps maintain a larger thermal gradient necessary for significant power generation. Recent works have shown promising advances in the optimization of TECs under externally controlled temperature gradients. For example, the addition of guanidinium cations (Gdm^+^) to selectively induce crystallization of [Fe(CN)_6_]^(4−)^ or the use of zwitterions to enhance the gel's mechanical stability along with the solvation of cyanide ions resulted in a great enhancement in the output current and reactivity of the system.^18–20^ Similarly, other optimizations of the electrolyte, *i.e.* the addition of ethanol^21^ and methanol^22^ to maintain a larger redox concentration gradient, have proven beneficial. Other improvements have been brought by the addition of a supporting electrolyte^23^ to exploit a stronger thermodiffusive contribution as well as the adoption of ionic liquids.^24–27^ Electrode modifications are also beneficial since they reduce the charge transfer resistance,^28,29^ and the use of carbon nanotubes has been shown to be particularly effective.^30,31^ Finally, synergistic exploitation of light and heat^32^ has shown great advances in the performance of the system. However, in all these improved solutions, an externally applied thermal gradient was imposed onto the system. Thus, hydrogels have not been studied in a passive cooling configuration, and the influence of different design parameters for an optimal power output is not fully understood.

This work addresses these gaps by demonstrating, for the first time, that hydrogel-based TECs (HyTECs) can operate effectively across a wide range of conditions. Notably, under passive cooling, where liquid-based TECs typically fail to provide a suitable response, HyTECs provide a stable and satisfactory performance. We show that HyTECs achieve power outputs of up to 3.5 μW cm^−2^ under the sole application of a hot temperature in the range of 55 to 65 °C. This performance is comparable to the state-of-the-art liquid-based TEC operation under an externally imposed thermal gradient of 10 K cm^−1^. We further report a comprehensive investigation of the thermoelectric performance of HyTECs under passive cooling conditions by analyzing the influence of a variety of critical parameters including redox-pair concentration and synergistic contribution of thermodiffusion and electrode spacing. Using a detailed Multiphysics COMSOL model, we attribute the unique behavior of HyTECs to their combined low thermal conductivity and permeability. These properties facilitate the formation of a stable convective cell within the device, enhancing the transport of the redox species and thus the produced current. We also demonstrate stable operation of HyTECs under passive cooling conditions for various orientations, with a minimal change in the output power from the horizontal position (hot surface at the bottom) up to an inclination of 135°. Overall, our work provides valuable insights and design guidelines for hydrogel-based TECs, paving the way for their application in real-world waste heat recovery.

## 2 Working principle and main contributions in thermoelectrochemical cells

**Fig. 1:**
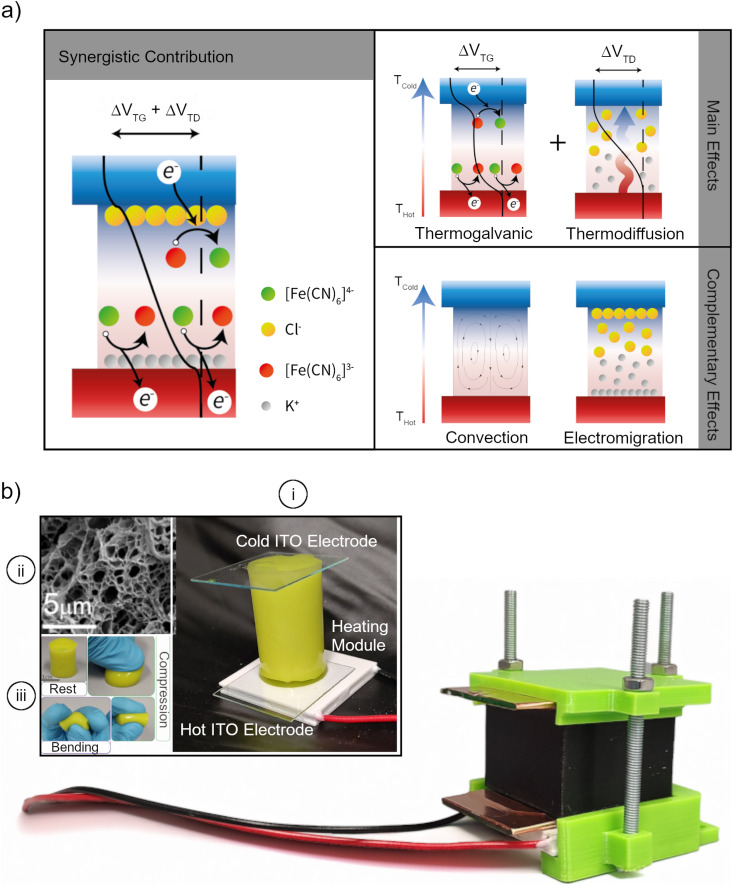
(a) Synergistic working mechanism and band structure of a HyTEC cell exploiting the main contribution given by redox pairs (thermogalvanic) and supporting electrolyte (thermoelectric) along with the complementary effect of convection and electromigration. (b) Optical image of the tightening cell containing the hydrogel electrolyte and the electrodes. To better show the structure, a bare image of the system is reported in panel (i) of the inset, while the SEM image of the freeze dried PVA hydrogel is reported in panel (ii) and the flexibility and resistance to deformation are showcased in panel (iii).

We developed a hydrogel-based thermoelectrochemical cell (HyTEC) composed of two identical indium–tin–oxide (ITO) electrodes sandwiching a polyvinyl alcohol (PVA) hydrogel. The hydrogel contains a ferro-/ferricyanide redox couple in aqueous solution and a potassium chloride (KCl) supporting electrolyte to regulate the ionic resistance of the system. PVA was chosen as the hydrogel matrix due to its relatively high swelling capacity (≈ 200%, see Fig. S1, ESI†), good mechanical properties, cost-effectiveness and simple preparation procedure. In this work, a physical cross-linking method has been adopted, specifically freeze-thawing (see Methods for more details). After the synthesis, the hydrogel was fully swollen in an aqueous solution containing potassium ferro-/ferri-cyanide and KCl. These redox couples are chosen because of the high reactivity and large entropy of the reaction difference between the two species.^33^ KCl was selected as the supporting electrolyte to maintain consistent cation concentrations and to leverage the strong thermodiffusion effects reported in recent studies.^23^ Additionally, KCl minimizes the impact on the cyanide reaction entropy at high concentrations. The performance of these HyTECs primarily depends on the interplay of thermogalvanic (TG) and thermodiffusion (TD) effects driven by the temperature gradient existing across the electrodes. These main effects are affected by the chosen redox species and the supporting electrolyte, respectively. A schematic of this effect is reported in Fig. 1a, along with the typical band alignment to underline their working principle. Electromigration and, importantly, natural convection further influence these processes (labeled as a complementary effect in Fig. 1a).^16,17,34^ More specifically, the TG effect is due to the temperature-dependent entropy of the reaction of the redox molecules that induces a thermogalvanic voltage (Δ*V*_TG_) and current (*I*_TG_).^23^ In our cell, ferricyanide is reduced to ferrocyanide at the hot electrode at a faster rate than ferrocyanide oxidation to ferricyanide at the cold electrode. This creates a concentration gradient of the redox species and a chemical potential gradient that transfers electrons from the hot to the cold sides, making TG the expected primary contribution for the TEC current. The TD effect, instead, is due to the migration of ions from the hot region to the cold region in response to the temperature gradient. We utilize KCl as a supporting electrolyte and thus, Cl^−^ (K^+^) ions accumulate near the cold (hot) electrode interface, generating a net potential difference (Δ*V*_TD_), which is strongly influenced by the temperature gradient and the selective diffusion of ions in the electrolyte.^35^ Electromigration arises in the system due to the electric field created by TG and TD, driving the migration of ions towards oppositely charged regions and increasing the achievable voltage.^36^ TD and electromigration also contribute to creating a distribution of ionic species, which gives rise to local variations in ionic conductivity.^23^ Natural convection instead has a two-fold impact on TG and TD. On one hand, it enhances heat transfer across the cell, reducing the available temperature gradient, which decreases the cell performance. On the other hand, it supplies fresh redox species at the electrodes, improving TG current and counteracting depletion of reactants that would stop the TEC.

Fig. 1b, presents an image of the compression cell used in the experiments, containing a hydrogel matrix that critically influences the heat and mass transport properties of the system. Transitioning from a liquid to a hydrogel electrolyte introduces a polymeric framework in the fluid path, which restricts the convective velocity of the fluid. However, the electrolyte within the hydrogel pores remains mobile, enabling sustained cell operation. Fig. 1b also includes an image of the HyTEC cell (panel i), a freeze-dried PVA hydrogel SEM image (panel ii), and the gel's response to mechanical stress (panel iii). Previous studies have highlighted the role of porosity in system behavior.^8^ Using MATLAB routines for SEM image analysis, we estimate an average pore size of 2 μm and an overall porosity of approximately 60% (Fig. S2, ESI†). Overall, the performance of a TEC is dictated by the interplay of all the above-mentioned effects, which becomes more complex when the system is operated under passive cooling conditions instead of under an externally fixed temperature gradient, due to the coupling between heat and mass transport in the system. In this study, we first demonstrate the key advantage of our HyTEC compared to a liquid-based TEC in terms of heat and mass transport under passive cooling conditions. Next, we optimize critical parameters such as the concentration of the redox pair, the supporting electrolyte and the hydrogel aspect ratio to maximize both the power density and efficiency of the HyTEC. Finally, we investigate the impact of cell orientation and assess the performance limits of HyTEC operation under real-world passive cooling conditions.

## 3 Role of hydrogels on heat and fluid transport (natural convection)

**Fig. 2:**
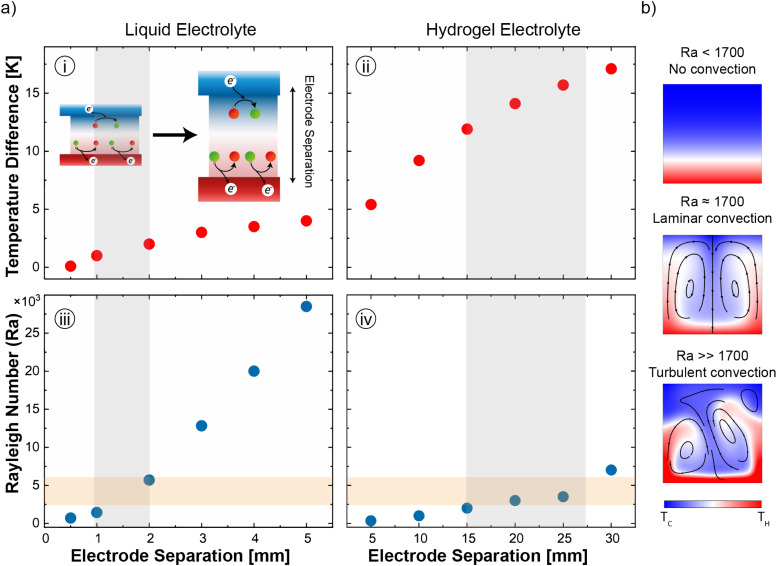
(a) Analysis by COMSOL simulation of (panels (i) and (ii)) the temperature gradient along with (panels (iii) and (iv)) the identification of the Rayleigh number in liquid-based and hydrogel-based thermoelectrochemical cells, as a function of the electrode separation. The shadowed lines indicate the region where laminar convection is occurring as the intersection between Ra number and electrode separation. The (panel (i)) inset explains the meaning of electrode separation in this framework. (b) Schematic of the fluid motion dependence on Ra number. The color map specifies the temperature distribution and the contours indicate the fluid flux, depicting the convection in the cell.

For any TEC, the larger the temperature gradient between its electrodes the higher the voltage output. For this reason, TEC performance is typically investigated under the application of an external temperature gradient. However, a passive cooling approach, *i.e.* based on the sole control of the hot electrode temperature, is more desirable for real world conditions, as it makes TECs widely applicable for waste-heat recuperation. This implies that the operating temperature gradient directly depends on the TEC heat and mass transport properties as well as its physical dimensions. Using a comprehensive COMSOL Multiphysics model (Fig. S3 with parameters reported in Table S1, ESI†), we compare the thermal behavior of a conventional liquid electrolyte and a hydrogel-based electrolyte as a function of the electrode separations, while keeping the lateral dimensions of the cell constant (width *W* = 10 mm and depth *D* = 10 mm). In all the simulations, the temperature of the hot electrode is set to 60 °C while the cold electrode is passively cooled *via* natural convection, allowing a realistic and dynamic temperature gradient to develop across the electrolyte. Fig. 2a clearly shows that a water-based electrolyte (see panel i) is not capable of developing and sustaining temperature differences higher than 5 K, regardless of its thickness, while a hydrogel-based electrolyte (see panel ii) can achieve larger temperature differences even for electrode separations as low as 5 mm. Thus, HyTECs are uniquely suited to operate in passive-cooling conditions. Since the thermal conductivity of the hydrogel (*k*_Hy_ ≈ 0.75 W m^−1^ K^−1^)^37,38^ is known to be slightly higher compared to that of the liquid electrolyte (*k*_L_ ≈ 0.6 W m^−1^ K^−1^), it is worth analyzing more closely the heat and mass transfer mechanisms in these cells to understand their behavior. The Rayleigh number (Ra) describes the ratio between the time scale of the thermal transport *via* diffusion compared to thermal transport *via* advection for a given temperature difference (Δ*T* = *T*_H_ − *T*_C_) and is given by:1
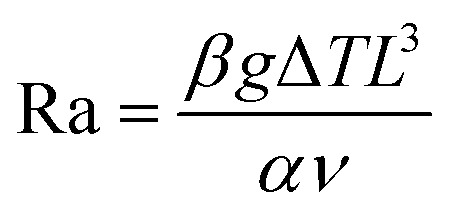
where *β* is the volumetric thermal expansion coefficient, *g* is the gravity, *L* is the thickness of the fluid layer, *ν* is the kinetic viscosity of the fluid and *α* is the thermal diffusivity.^39^ As shown in Fig. 2b, the fluid motion in a horizontal cell with a heated bottom surface is highly dependent on the Ra value. Lower Ra values result in a purely diffusive heat transport, which corresponds to a stratified fluid (Fig. 2b, top), while higher Ra values translate into turbulent flows with complex flow patterns (Fig. 2b, bottom). Based on our COMSOL Multiphysics model, stable laminar convective cells are developed for 1708 < Ra < 5 × 10^3^ (Fig. 2b, middle). The upper limit of the Ra number for convective flow within the cell is an order of magnitude lower than values reported in the literature.^40^ However, studies have shown that turbulence in convection within liquid systems can begin at lower Rayleigh numbers due to a number of factors such as medium heterogeneities, nonlinear flow effects (*e.g.*, Forchheimer and Brinkman corrections), liquid properties like low viscosity and high thermal expansion, and geometric factors such as confined domains and uneven boundary conditions, which collectively amplify instabilities.^41,42^

Importantly, the flow behavior dramatically impacts the transport and concentration of the redox flow and ionic species in the electrolyte, directly influencing the TEC power output. In panels iii and iv of Fig. 2a, Ra was calculated as a function of the electrode separation for both electrolyte systems. To obtain the Rayleigh number in the case of a hydrogel-based electrolyte, we adopted the following expression:2
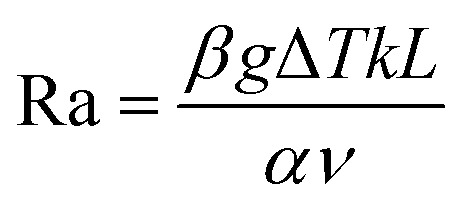
where the flow in porous media is accounted for in the parameter *k* representing the fluid permeability.^42^ The orange shaded region represents the Ra range where transition between the laminar and turbulent flow is expected to occur. The liquid electrolyte exhibits Ra values that increase with the electrode separation, reaching Ra > 5000 after just 2 mm. This confirms the rapid transition from a conduction-dominant heat transfer to turbulent convection. While enhancing mass transport, this regime greatly decreases the temperature gradient and leads to inefficient energy conversion.^39^ Contrarily, the hydrogel-based electrolyte system maintains Ra values well below the critical threshold, even at larger electrode separations. This suggests that while mass transport is limited, stability is achieved for both convective motion and temperature gradient within the system. This stability is critical for sustaining the TG and TD contributions to the cell's output power.

These results highlight the significant advantage of using hydrogel-based electrolytes over conventional liquid electrolytes in passively cooled TECs, extending beyond improvements in safety, handling and operational reliability. HyTECs can sustain larger and more stable temperature gradients under the application of only the hot temperature. Additionally, they benefit from stable flow recirculation, which enhances the redox reaction rates and maintains their concentration gradient, boosting the overall TEC performance.^32^

## 4 Role of the redox pair and supporting electrolyte concentrations on the power output

Having established the role of hydrogels and outlined their benefits, we next individually assess the role of the redox pair and electrolyte concentrations, including their distinct contributions to the TG and TD effects. To experimentally test the response of our HyTEC we used two ITO electrodes (of 100 nm thickness deposited on fused silica wafers) and designed a compression cell to securely hold them, ensuring a good interface with the electrolyte (see Fig. 1b). In a typical experiment, the heat source is mimicked using a Peltier module connected to a power supply to simulate different temperature conditions. For all the measurements, hydrogel samples of 6 mm thickness and 10 × 10 mm area were used. Additionally, a comparison with a sample of 4 mm thickness is provided while investigating the effect of the supporting electrolyte. The voltage and current are measured using a potentiostat in a two-electrode configuration, while the temperature across the sample is monitored with a pair of calibrated type K thermocouples, and a data logger is used for data acquisition.

**Fig. 3:**
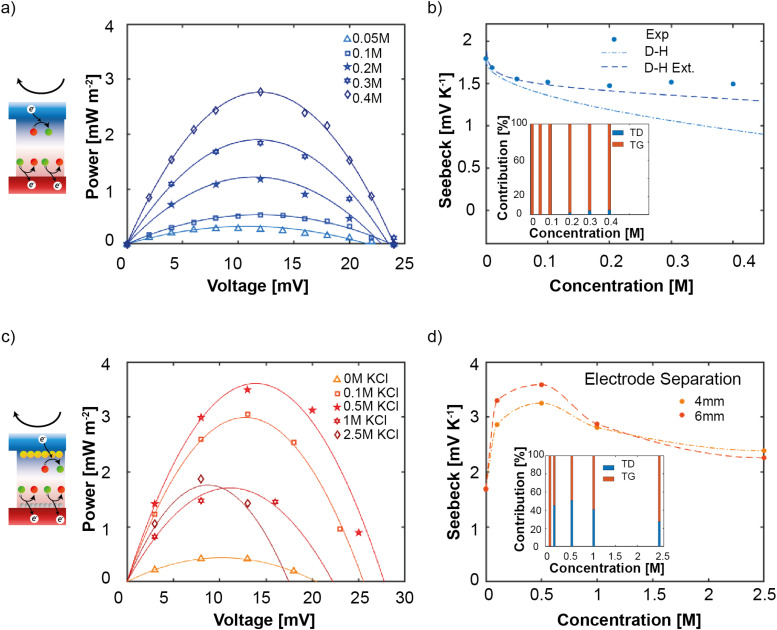
Schematics of the experimental situation analyzed using a hot source at *T*_Hot_ = 60 °C and a sample thickness of 6 mm with (a) no supporting electrolyte and (c) redox + supporting (KCl) electrolyte. The power output as a function of voltage obtained using LSV is reported right next to the schematic. The Seebeck coefficient is calculated in panels (b) and (d), with the latter showing the comparison between two different sample thicknesses. The insets to these plots represent the calculated thermodiffusive (TD) and thermogalvanic (TG) contributions to *V*_oc_.

First, we consider the performance of the HyTEC without any supporting electrolyte. The power–voltage curves as a function of the ferrocyanide/ferricyanide concentration for a hot source temperature *T*_Hot_ = 60 °C are shown in Fig. 3a. In all cases, a stoichiometric 1 : 1 ratio of ferro-/ferri-cyanide species was used, as according to the Butler–Volmer theory this ratio maximizes the power output (Fig. S4c, ESI†).^43^ Furthermore, we note that 0.4 M is the highest possible concentration due to the solubility limit of these redox species.^44^ At low concentrations, the experimental current density is limited by the low availability of redox species, leading to low reaction rates at the electrodes and thus a low peak power output (blue curve). As the number of reactive species available increases, the power density also increases and a linear relation with concentration is obtained (Fig. S4a and b, ESI†). On the other hand, we observe that the open circuit voltage (intersection with the *x*-axis) is not significantly impacted by the redox species concentration, in agreement with the Debye–Hückel theory.^43^

From these curves, the Seebeck coefficient of each HyTEC can be obtained as:3
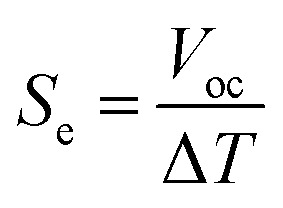


Fig. 3b shows a comparison of the experimental (blue dots) and theoretical (lines) Seebeck coefficients. The dashed-dotted line corresponds to the Debye–Huckel theory^45^ that ignores interactions between ions, while the dashed curve is an extended model that partially includes this effect *via* the introduction of an ion-to-ion distance.^46^ We observe that the Seebeck coefficient initially decreases with concentration and then plateaus for high concentration values. Additionally, a slight deviation between experiments and theory persists even for the extended model. Interestingly, as TD is entirely neglected in the theoretical approach and is expected to play an increasing role with the redox species concentration, we attribute such a deviation to the TD contributions. This analysis enables us to estimate the TG and TD contributions to the output voltage for each HyTEC (see Section S1 in the ESI†). As shown in the inset of Fig. 3b, the TD contribution is negligible for redox species concentration below 0.2 M and increases to ≈ 5% for 0.4 M.

Next, we investigate the role of the supporting electrolyte. The appropriate selection of the supporting electrolyte concentration ensures that the ionic conductivity is maximized without introducing diffusion limitations. Moreover, the interaction of the supporting electrolyte with water molecules is an important parameter to account for, as it can limit the ion mobility. Based on previous studies, potassium chloride is chosen as a preferred salt for this analysis^23,47^ and we vary its concentration up to 2.5 M. Based on the previous discussion, we instead fix the concentration of redox species to 0.1 M (1 : 1) in order to neglect their TD contribution. Fig. 3c shows the dependence of the power density as a function of voltage for different KCl concentrations. We immediately notice the remarkable increase in both the open-circuit voltage and maximum power output when just 0.1 M KCl is added to the electrolyte (triangles *versus* squares). We further observe that 0.5 M KCl maximizes the cell performance (stars). This is an indication of the importance of supporting electrolytes and the contribution given by thermodiffusion. Interestingly, the Seebeck coefficient exhibits a large change with the KCl concentration, due to the accumulation of K^+^ and Cl^−^ at the electrode/electrolyte interface *via* TD. In particular, *S*_e_ shows an increase of ≈100% at around 0.5 M KCl concentration (Fig. 3d), with Δ*V*_TD_ accounting for nearly 50% of the output voltage in this regime (inset).

Furthermore, in the 0.1–0.5 M concentration range, we observe that *V*_oc_ shows only a modest improvement while the maximum power output shows a dramatic increase (from 0.4 W m^−2^ to 3.6 W m^−2^). This can be attributed to the enhanced ionic conductivity of the electrolyte. In fact, by reducing the overall ohmic resistance of the system, the cell supports much higher current densities, limiting energy losses. Moreover, the addition of KCl reduces polarization losses, including both ohmic and concentration polarization, which improves the efficiency of the redox reactions at the electrodes.^48^ In fact, the use of supporting electrolytes improves mass transport by minimizing concentration gradients of ferrocyanide and ferricyanide ions at the electrodes, helping to maintain a stable redox reaction rate.^16^ This enhancement of mass transport reduces ion depletion near the electrodes, further contributing to an increase in the power output.

By increasing the concentration of the supporting electrolyte beyond 0.5 M, a decrease in both the Seebeck response and the power output is observed (Fig. 3c and d). This is likely related to a reduction in the electrochemical gradient as the ion concentration difference between the electrode/electrolyte interface and the bulk solution decreases, diminishing the driving force for the electrochemical reaction and the ion transport. Moreover, even though an increase in the electrolyte concentration leads to lower resistance, in principle resulting in higher currents, the voltage drops due to the screening of the ions and dilution of active species.^49^ In TEC, these two are intimately bound and a lowering of *V*_oc_ inevitably translates into a drop in the reaction rate. As a final consideration, at very high electrolyte concentrations, the electric double layer (EDL) is highly compressed due to strong ionic screening, resulting in a reduced Debye length.^50,51^ The diffusion layer becomes thinner due to enhanced ionic availability, but increased solution viscosity and ion–ion interactions, such as ion pairing and clustering, which can reduce ion mobility, reducing the contribution to Δ*V*_TD_. This is particularly evident in Fig. 3d inset, where the TD contribution is observed to decrease below 30%.

## 5 Optimized HyTEC design and performance

Based on the previous analysis, we set the optimal concentrations of the ferro/ferricyanide redox species and the supporting KCl electrolyte concentrations to 0.4 M and 0.5 M, respectively. Following our initial discussion, we then investigate the HyTEC performance as a function of the electrode separation, *i.e.* the hydrogel thickness, to evaluate how natural convection within the cell influences the system response. Previous studies based on an externally applied temperature gradient showed negligible influence of the electrode separation on the TEC power output.^52–55^ However, under passive cooling conditions, the temperature difference across the cell increases with larger electrode separation (Fig. 2b). Thus, stronger dependence of the power output on this parameter is expected (as can be seen in Fig. S5 and S6, ESI†).

**Fig. 4:**
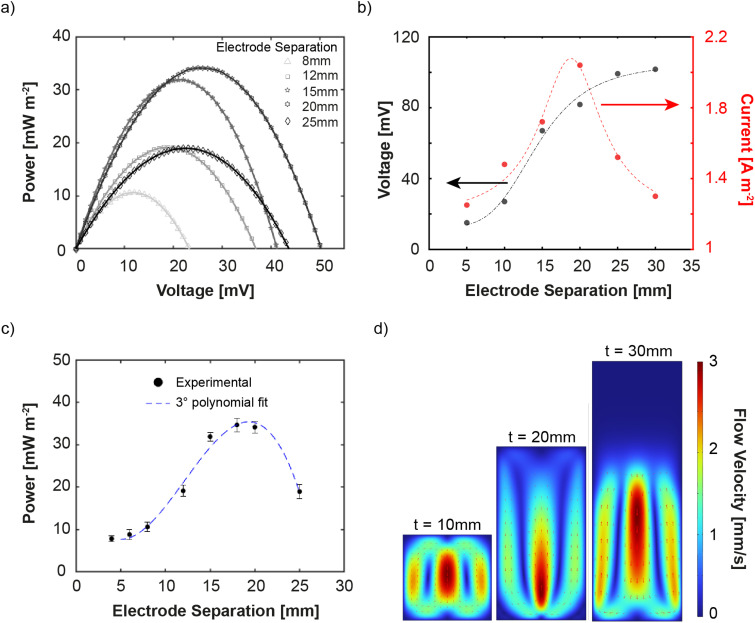
(a) The output power measured experimentally for TEC cells with different electrode separation (*i.e.* electrolyte or hydrogel thickness) as a function of voltage. (b) *V*_oc_ and current density calculated with a complete COMSOL multiphysics simulation as a function of electrode separation. (c) The experimental result for the maximum output power as a function of electrode separation along with a 3rd-order polynomial fit just to underline the trend. (d) The convection velocity at the electrode/electrolyte interface obtained for cells with 10 mm width and different heights, explaining the reason for the performance decay after 20 mm (obtained from simulations).

**Fig. 5:**
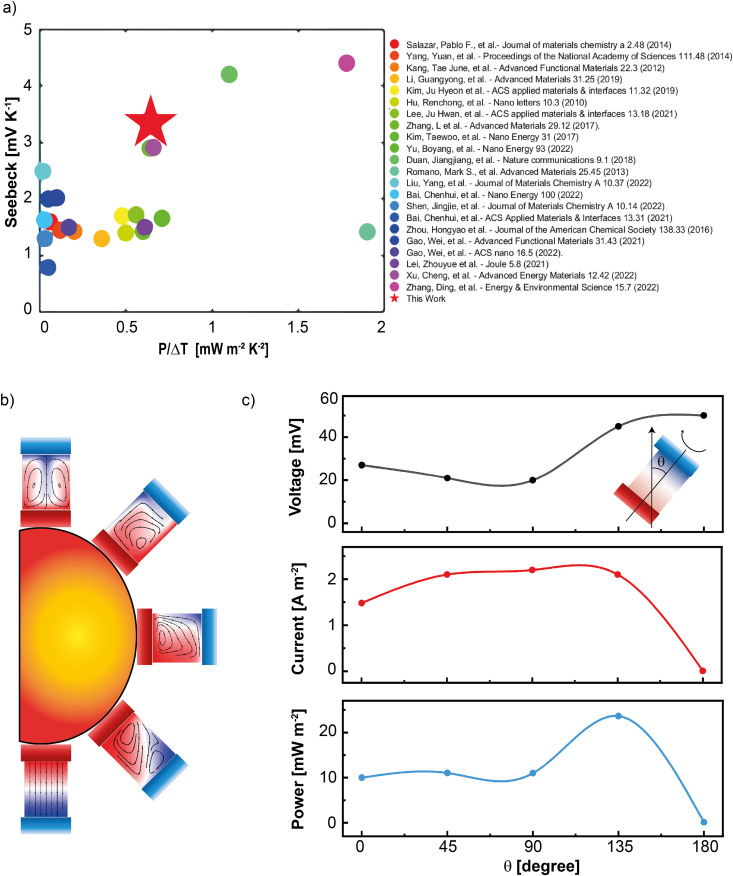
(a) Comparison of the normalized performance (power output per temperature gradient) of this work (red star) with previously reported TECs from the literature. (b) Schematic of the HyTEC on a hot pipe as a use case scenario. (c) Performance of the cell in terms of *V*_oc_, *I*_sc_ and *P*_max_ reported as a function of the orientation angle (*θ*).

Fig. 4a shows the HyTEC experimental power–voltage curves for electrode separation increasing from 8 mm up to 25 mm. Interestingly, a non-monotonic behavior of both *V*_oc_ and peak power output is observed. Notably, the peak power output increases by ≈ 400% when going from an electrode separation of 5 mm to 20 mm (Fig. 4c). However, with a subsequent increase in electrode separation, the output power declines. This trend is due to the balance between building a higher temperature gradient across the cell and enabling efficient ion transport, demonstrating that while some separation is necessary to sustain a sufficient temperature gradient, excessive spacing leads to performance losses due to inefficient ion transport. To identify the main factor driving this trend, we rely on the COMSOL Multiphysics model. Fig. S8 (ESI†) shows excellent agreement with the experimental results, including a rapid increase in the power output of up to 20 mm electrode separation and a decrease afterwards. Fig. 4b further shows the calculated *V*_oc_ and the short circuit current density. We notice that *V*_oc_ (grey curve) saturates for electrode separations larger than 25 mm, in agreement with a saturating temperature difference trend reported in Fig. S6 (ESI†). On the other hand, the short circuit current density (red curve) peaks at approximately 20 mm and dramatically decreases at larger separations. Thus, the electrode separation has a significant impact on the coupling of the thermal and electrochemical responses of the HyTEC, with a predominant influence of the generated current. To unravel why the short-circuit current peaks at around 20 mm electrode separation, we examine the convection velocity in the HyTEC cells as a function of electrode separation (Fig. 4d). For small electrode separations, thermal energy is sufficient to form a stable convective cell extending throughout the entire HyTEC. An increase in electrode separation translates into a more developed convective profile, with better mass transport and faster velocities close to the electrode/electrolyte interface. However, when the electrode separation becomes too large, the thermal energy is not sufficient to form a convective cell that extends throughout the HyTEC. As a result, the flow velocity at the electrode/electrolyte interface becomes negligible, lowering the performance of the system since the main ion transport mechanism becomes electro-migration instead of advection. Overall, our optimized HyTEC with 20 mm electrode separation exhibits a power output of 35 mW m^−2^ under passive cooling conditions (60 °C of the hot surface and 21 °C of the environment). This corresponds to a Seebeck coefficient of 3.5 mV K^−1^ (see Fig. S7, ESI†) and a normalized power of 0.6 mW m^−2^ K^−2^ in line with state-of-the-art TEC systems previously tested under externally applied temperature gradients, as can be observed in Fig. 5a.

Concerning the device performance in general, it has to be said that great progress in the output power enhancement has been made recently. Studies have demonstrated that a variety of material and cell-architectural strategies can drive thermogalvanic Seebeck coefficients into the tens of mV K^−1^. For example, polymeric or gel electrolytes with strong ion confinement or specific redox interactions have yielded very high voltages: an oxidized cellulose membrane confining Na^+^ ions achieved ≈ 24 mV K^−1^ (ref. 56) and a gelatin–salt gel combining KCl/NaCl thermodiffusion with an Fe(CN)_6_^4−/3−^ redox reached ≈ 1 mV K^−1^.^23^ Similarly, thermoresponsive polymers (*e.g.* methylcellulose) have been used to switch electrolyte polarity and amplify redox concentration gradients, dramatically boosting both the entropy change and the concentration difference of the I^−^/I_3_^−^ couple.^35^ Asymmetric or phase-changing electrodes are another key approach: for instance, Shin *et al.* exploited a Na–K alloy electrode that melts at a controlled temperature, sharply increasing the redox entropy at one interface and giving ≈ 26 mV K^−1^.^57^ Finally, photo- or nanoparticle-enhanced systems create continuous redox gradients or catalytic effects: *in situ* photocatalytic H_2_/O_2_ evolution maintained a steady concentration difference in an iron-based cell, yielding ≈ 8 mV K^−1^ under illumination.^32^ Likewise, use of non-aqueous media (ionic liquids or mixed solvents) can further tune solvation entropy to raise Seebeck coefficients.^14^

Eventually, to assess the potential of our HyTEC for real-world waste heat recovery, we use simulations to understand the orientation dependence on the performance of the HyTEC system. A practical application scenario involves surrounding a hot pipe with HyTECs.^58^Fig. 5b provides a schematic depiction of this situation, with solid black lines representing fluid flow and color maps illustrating the temperature profile for each orientation. These schematics are obtained using COMSOL simulations, considering a 10 × 10 × 10 mm HyTEC with optimized design on a hot surface at 60 °C, with the orientation angle (*θ*) being the only variable. We specifically chose the 10 × 10 × 10 mm cell to avoid any effect of cell dimensions on performance, as our focus here is to study the effect of gravity. Changes in thermal distribution and fluid dynamics, driven solely by gravitational forces, profoundly affect device performance. Fig. 5c shows the calculated *V*_oc_ (black line), short circuit current density (red line) and maximum power density (blue line) as a function of the orientation angle (*θ*), defined with respect to an upward-pointing *z*-axis as shown in the inset. The data points correspond to the *θ* values represented in panel (b).

Consistent with previous observations,^58,59^ the 135° orientation exhibits the highest power output. This configuration offers an optimal trade-off between preserving a sufficient temperature gradient and enabling moderate convective transport. Although the 180° orientation (*i.e.*, hot-above-cold) best suppresses natural convection and therefore maintains the largest sustained Δ*T*, the resulting electrolyte is essentially stagnant. Without fluid motion, redox species near the electrode surfaces are rapidly depleted, and the thermogalvanic current decays. At the other extreme, the 0° configuration (hot-below-cold) induces strong convective loops, which efficiently replenish the redox species at the electrodes but also promote heat mixing, thereby reducing Δ*T* and limiting the open-circuit voltage. The 135° tilt provides an intermediate regime: convection is partially suppressed, preserving a higher Δ*T* value than in the 0° case, while still allowing gravity-driven flow along the cell walls to circulate redox species to the electrode surfaces. This combination leads to a steady-state enhancement in both voltage and current, resulting in peak power output. This interpretation is supported by our COMSOL simulations, which show persistent but gentle internal flow at this tilt, and is consistent with prior literature findings. Overall, we see that the HyTEC device performs well from 0° to 150° under passive cooling conditions, which is promising for real-world waste heat recovery applications. Notably, we also observe similar trends for cells with different electrode spacing, reinforcing the robustness of these findings across different cell configurations.

## 6 Conclusions

This study presents a significant advancement in the field of thermoelectrochemical cells (TECs) by demonstrating the robust performance of hydrogel-based TECs (HyTECs) under passive cooling conditions for a wide range of operating scenarios. Unlike liquid-based TECs, which often fail to provide satisfactory performance in the absence of externally imposed thermal gradients, HyTECs achieve stable operation solely through the application of a hot temperature. Central to the exceptional performance of HyTECs is their polyvinyl alcohol (PVA) hydrogel electrolyte, which combines ferro-/ferricyanide redox couples with a potassium chloride supporting electrolyte. The porous structure of the hydrogel improves ionic transport while minimizing undesirable convective disturbances. By optimizing the interplay of thermogalvanic, thermodiffusion, and convection effects, HyTECs leverage the unique properties of the hydrogel matrix – namely, low thermal conductivity and permeability – to facilitate stable convective cells within the device. This stability enhances the transport of redox species, boosting redox reaction rates and current generation. A comprehensive investigation of HyTECs under passive cooling conditions revealed critical insights into the influence of various design and operational parameters. Redox pair concentrations, for example, play a crucial role in enhancing power density, as higher ferrocyanide/ferricyanide concentrations increase redox reaction rates. Similarly, the supporting electrolyte (KCl) improves ionic conductivity and thermodiffusion, with an optimal concentration of approximately 0.5 M, reducing resistance and polarization losses while enhancing performance. However, excessive KCl concentrations lead to performance degradation due to ionic screening effects and diminished thermodiffusion contributions, highlighting the importance of careful electrolyte optimization. The electrode separation, or hydrogel thickness, emerged as another key parameter influencing HyTEC performance. Unlike liquid-based TECs, which show negligible dependence on electrode spacing under externally applied temperature gradients, HyTECs benefit significantly from larger separations under passive cooling. Experimental and computational analyses revealed that increasing the electrode separation up to 20 mm results in a dramatic ≈ 400% increase in power output, attributed to the enhanced temperature gradient and stable convective motion within the cell. Beyond 20 mm, however, performance declines due to insufficient thermal energy to sustain convection, emphasizing the need for a balance between the temperature gradient and ion transport efficiency. With an optimized design featuring 20 mm electrode separation and concentrations of 0.4 M ferro/ferricyanide and 0.5 M KCl, HyTECs achieve a power output of 35 mW m^−2^ under passive cooling conditions. This corresponds to a Seebeck coefficient of 3.5 mV K^−1^ and a normalized power of 0.6 mW m^−2^ K^−2^, rivaling the performance of state-of-the-art TEC systems operating under controlled thermal gradients. These results underscore the potential of HyTECs for efficient and scalable energy conversion in practical waste heat recovery scenarios. Simulations further highlight the versatility of HyTECs in real-world applications, such as waste heat recovery from hot pipes. The orientation of the device significantly impacts performance, with a 135° configuration yielding the highest power output due to improved temperature gradients and convective motion. Importantly, HyTECs demonstrate stable operation across a broad range of orientations (0°–150°), ensuring reliable performance under various installation conditions. In conclusion, this work provides valuable insights into the design and optimization of hydrogel-based TECs, paving the way for their integration into practical waste heat recovery systems. By addressing critical gaps in TEC technology, HyTECs emerge as a promising solution for sustainable energy generation, offering not only performance comparable to state-of-the-art systems but also enhanced safety, reliability, and ease of handling. This study lays the foundation for further exploration of hydrogel-based electrolytes and their potential to revolutionize thermoelectrochemical applications in diverse energy contexts.

## Conflicts of interest

There are no conflicts to declare.

## Supplementary Material

MH-012-D5MH00771B-s001

## Data Availability

All data supporting this study will be made available in a repository on Zenodo and provided with a corresponding DOI. Additional information and supporting data are provided in the ESI.†
