# Supplementary material for: Hydrogel-based thermoelectrochemical cells for waste heat recovery under passive cooling conditions
Source: Mater Horiz. 2025 Jul 2;12(18):7594–605. doi: 10.1039/d5mh00771b (PMC12219540; doi:10.1039/d5mh00771b)
Supplement: MH-012-D5MH00771B-s001 [file MH-012-D5MH00771B-s001.pdf]

Supplementary Information (SI) for Materials Horizons.
This journal is © The Royal Society of Chemistry 2025

Hydrogel-Based Thermoelectrochemical Cells for Efficient Waste Heat Recovery Under Passive Cooling Conditions

Authors: Matteo Bevione, Narmada Gopal Naidu, Giulia Tagliabue

Supporting Information

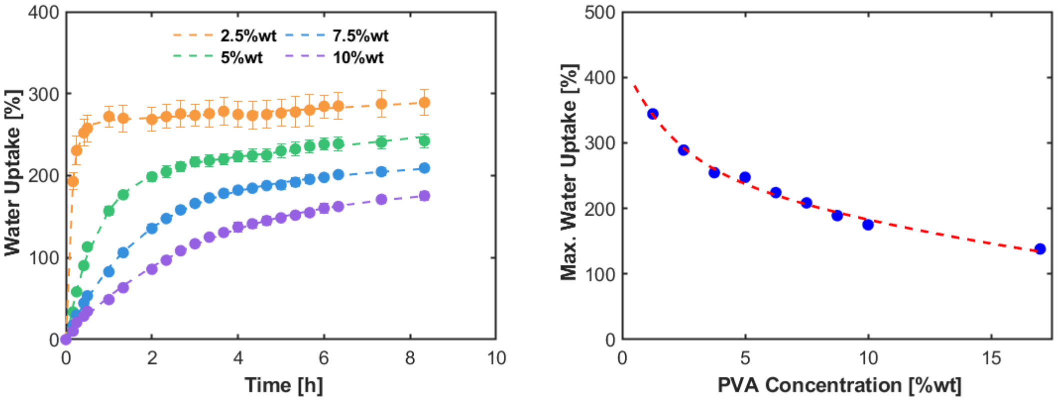

Figure S1: Polyvinil alcohol hydrogel swelling behavior: a) Water uptake in time using different weight concentration of PVA and b) maximum water uptake vs PVA concentration.

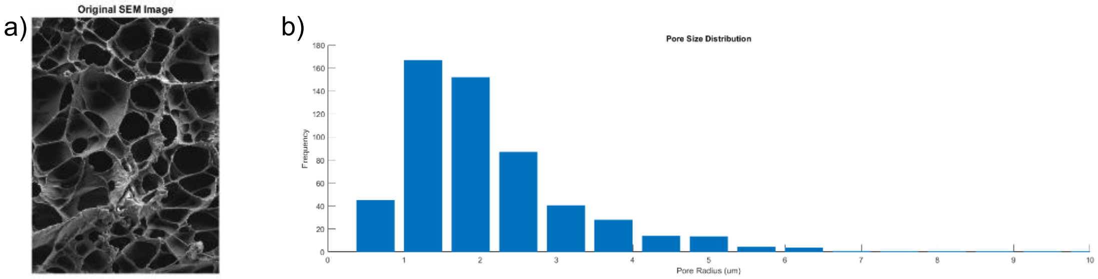

Figure S2: a) SEM image of the freeze dried PVA hydrogel and b) Pore size distribution obtained using MATLAB routine.

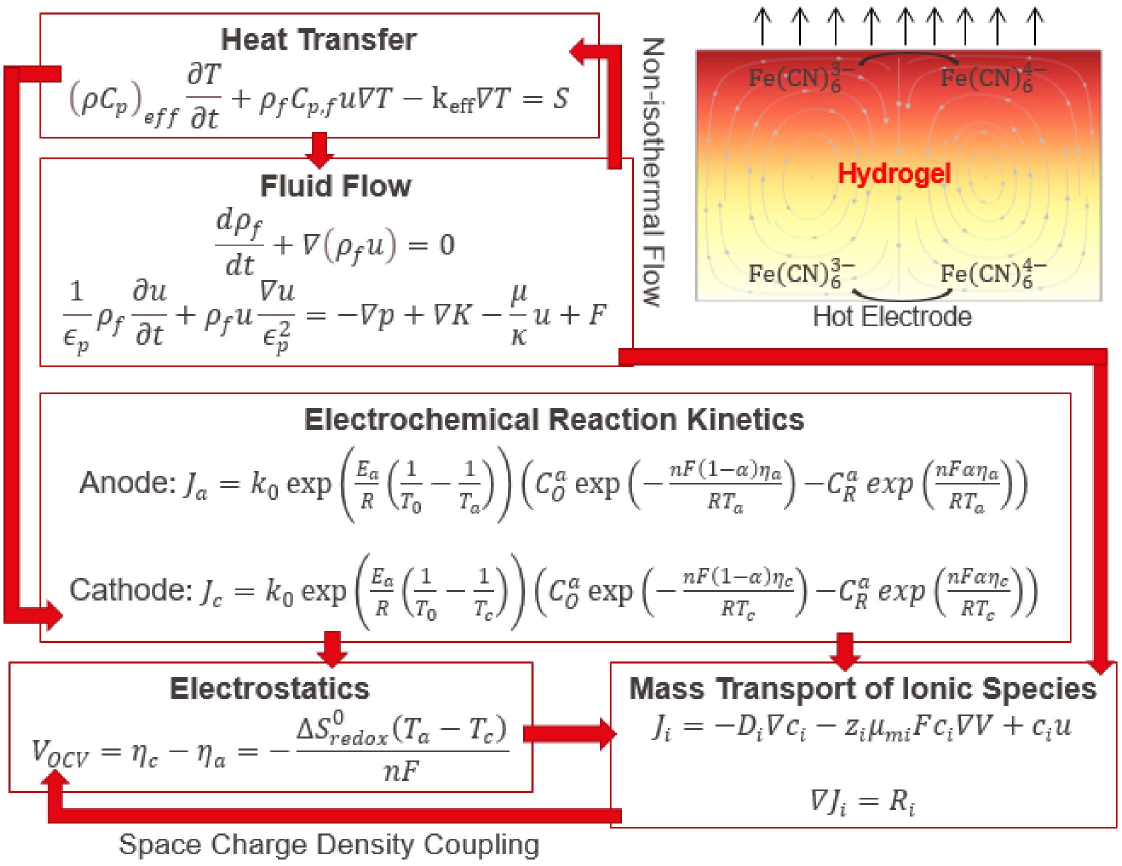

Figure S3: Structure of the COMSOL simulation of a TEC

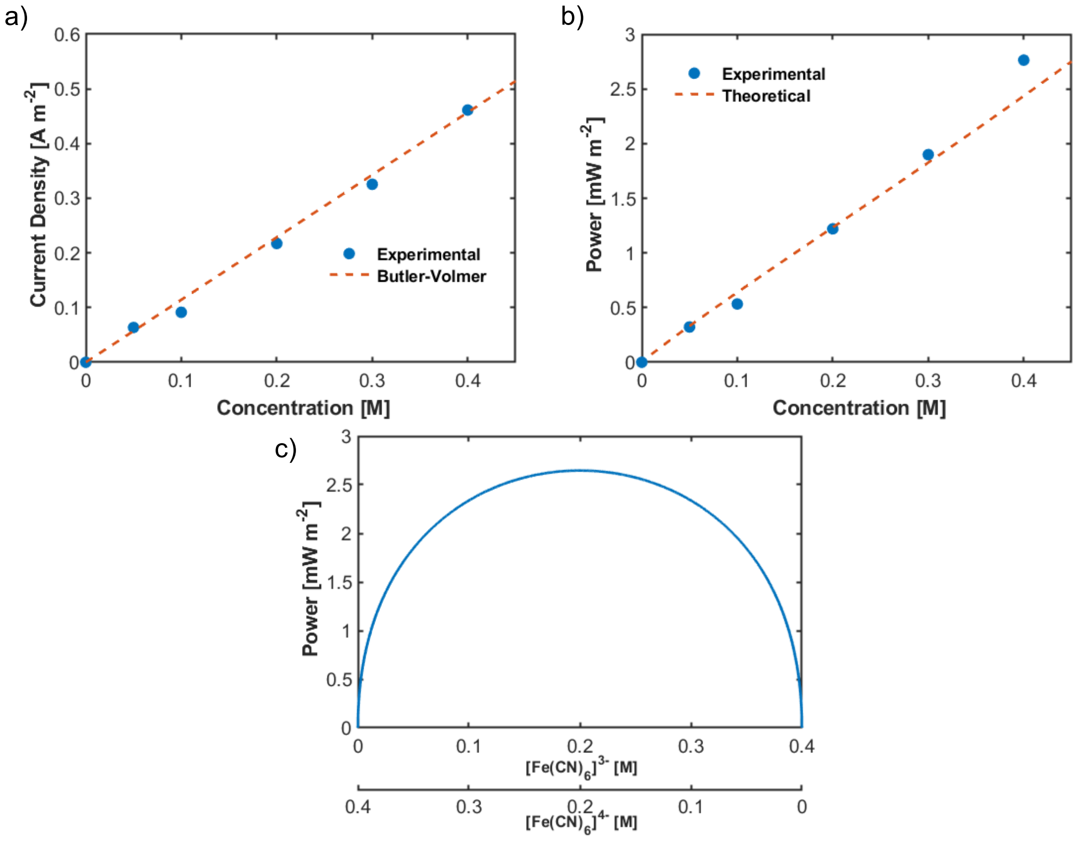

Figure S4: a) Current and b) power density measured experimentally (blue dots) as a function of concentration along with the behavior theoretically predicted (dash line) by Butler-Volmer theory. c) Theoretical prediction of power output as a function of the stoichiometric ratio between ferro- and ferri- cyanide.

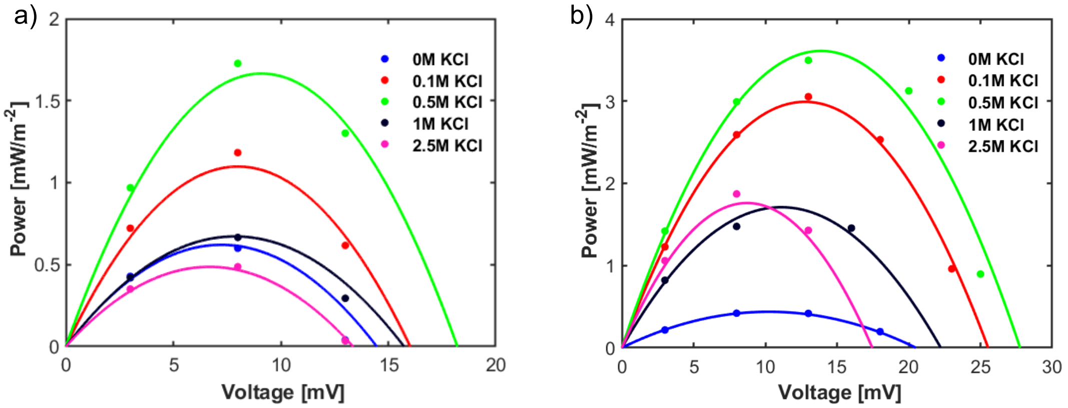

Figure S5: Output power as a function of supporting electrolyte (KCl) concentration for samples with thickness a) 4mm and b) 6mm.

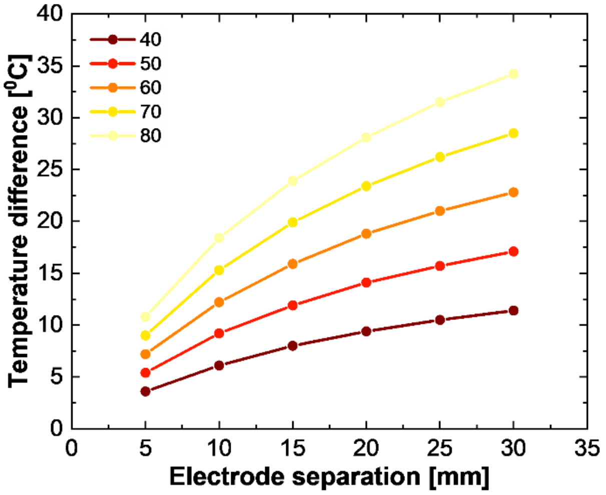

Figure S6: Temperature difference trend across hydrogel obtained using COMSOL simulation as a function of electrode separation. The tempearure reported in the legend regard the hot side of the cell.

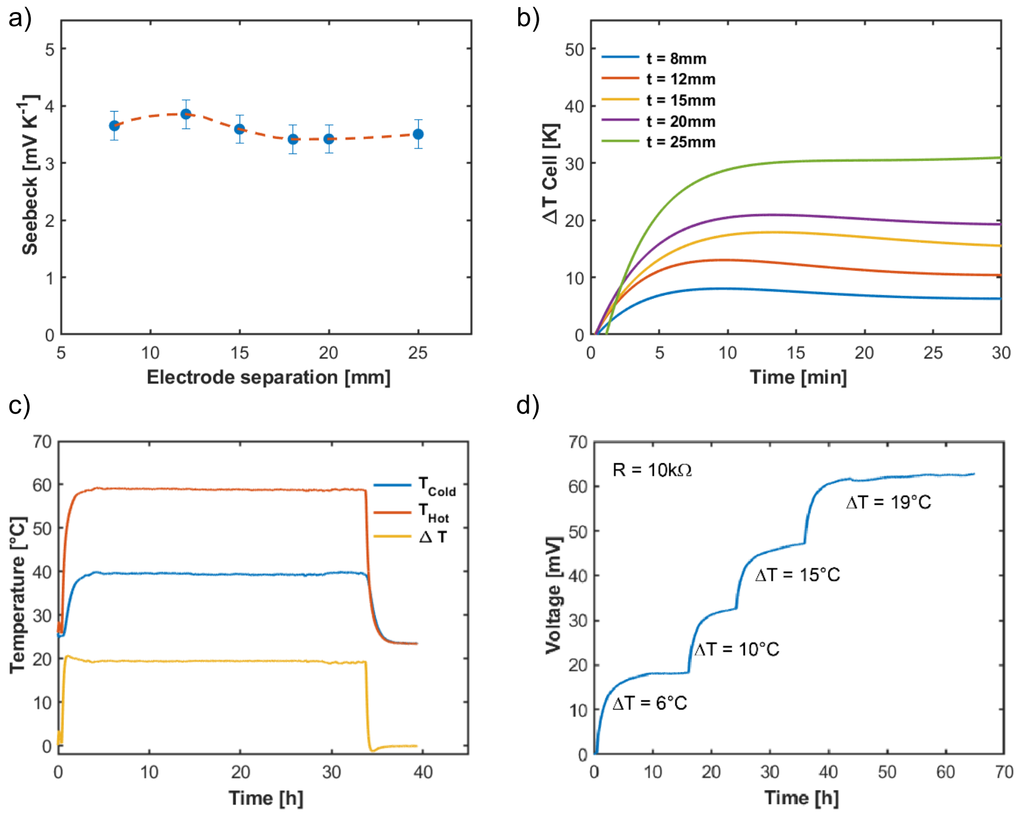

Figure S7: a) Seebeck coefficient experimetally obtained at the different electrode separation. b) Temperature difference recorded in time across the HyTEC cell. c) Temperature difference recorded maintaining the hot side ar 60°C for more than 30h to show stability upon passive cooling. d) Voltage measured across a $10k\Omega$ applying different temperature at the hot side of the cell. On the graph are reported the temperature differences across cell.

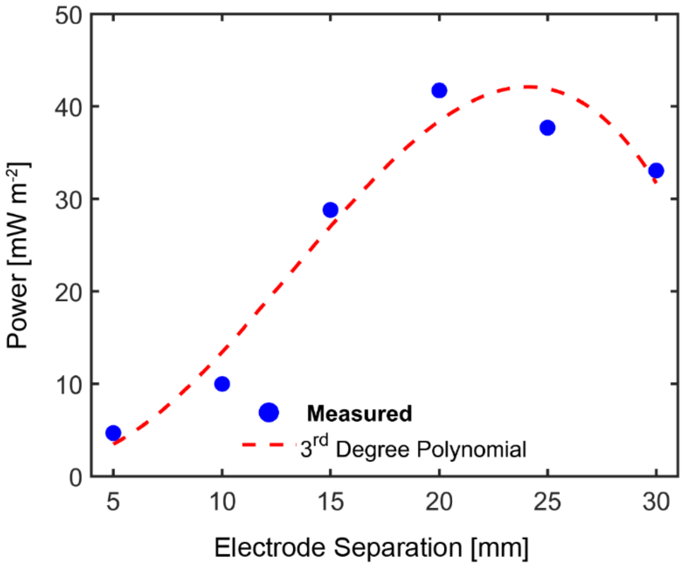

Figure S8: Values of power computed with the COMSOL simulation for different values of electrode separation (blue dots) along with a 3rd degree polynomial fit.

Table S1: Parameters used in the COMSOL simulation

Parameter	Value
Cell width (mm)	10
Cell height/electrode separation (mm)	5, 10, 15, 20, 25, 30
Density of hydrogel (kg m^{-3})	1190
Density of electrolyte (kg m^{-3})	$\rho=1063+0.546\times T-0.00147\times T^2$
Heat capacity of hydrogel (J kg^{-1} K^{-1})	1550
Heat capacity of electrolyte (J kg^{-1} K^{-1})	$12010.147-80.407\times T+0.309\times T^2-5.382\times 10^{-4}\times T^3+3.625\times 10^{-7}\times T^4$
Thermal conductivity of hydrogel (W m^{-1} K^{-1})	0.8
Thermal conductivity of electrolyte (W m^{-1} K^{-1})	$-0.869+0.009\times T-1.583\times 10^{-5}\times T^2+7.975\times 10^{-9}\times T^3$
Porosity of hydrogel	0.6
Hydraulic Permeability (m^2)	10^{-6}
Coefficient of thermal expansion of electrolyte (K^{-1})	$-1/\ \rho(T)\times d\rho(T)/dT$
Dynamic viscosity of electrolyte (Pa.s)	$1.379-0.021\times T+1.360\times 10^{-4}\times T^2-4.645\times 10^{-7}\times T^3+8.904\times 10^{-10}\times T^4-9.079\times 10^{-13}\times T^5+3.845\times 10^{-16}\times T^6$
Charge transfer coefficient	0.5
Number of electrons transferred	1
Anode temperature (K)	323
Heat transfer coefficient (W m^{-2} K^{-1})	30
Ambient Temperature (K)	293.15
Initial $K_3Fe(CN)_6$ concentration (mol m^{-3})	200
Initial $K_4Fe(CN)_6$ concentration (mol m^{-3})	200
Diffusion coefficient (m^2 s^{-1})	$(62.655-0.533\times T+1.1482\times 10^{-3}\times T^2)\ \times 10^{-10}$
Rate constant (ms^{-1})	$1.68\ x\ 10^{-6}$
Thermal-to-voltage conversion (mV K^{-1})	1.5

S1. Calculate thermodiffusive contribution

The contribution to thermovoltage given by the redox reaction and the thermodiffusion is computed starting from the argument that the deviation from Debye-Huckel model observed in Figure 2 is attributable to thermodiffusion. This hypothesis rises from the observation that at low concentration regimes, this effect is negligible and dominated by the redox activity. Moreover, the model only represents the redox activity assuming no thermodiffusion is occurring. This is not the case when the amount of salts in the electrolyte is such to generate a non-negligible accumulation at the electrode-electrolyte interface. Thus, calling the measured thermovoltage as S_{exp} and the one given by the model as S_{DH} we can get the contribution of thermodiffusion S_{TD} as the difference between these two. Thus the TD contribution (C_{TD}) can be expressed as

$$C_{TD} = \frac{S_{exp} - S_{DH}}{S_{exp}}$$

While the TG one is $C_{TG} = 1 - C_{TD}$.

Similar argument can be given for the addition of supporting electrolyte, where no chemical reaction are expected, thus contributing effectively only to the voltage generation. However, here we need to rely on the theoretical value given by DH theory ($S_{DH}^{\{Red\}}$) given for redox pairs with the measured deviation from this value being the thermodiffusive contribution of supporting electrolyte (SE), i.e.

$$C_{TD}^{SE} = \frac{S_{exp}^{SE} - S_{DH}^{Red}}{S_{exp}^{SE}}$$

S2. Thermodynamic Efficiency Limit of TECs

Generally, we can say the theoretical thermodynamic efficiency limit for TECs is governed by a Carnot efficiency:

$$\eta_{Carnot} = 1 - \left(\frac{T_{cold}}{T_{hot}}\right)$$

This means that approximately the theoretical limit is around 10% assuming a temperature difference similar to the experimental one, i.e. about of 30°C. However, real TEC efficiency is lower because of losses at the electrode-electrolyte interface, resistance to ion diffusion in the electrolyte, and the ability to maintain a stable gradient across the electrolyte. In literature, the Carnot relative efficiency of TEC system is expressed as:

$$\eta_r = \frac{\eta}{\eta_C} = \frac{\frac{P_{Max}}{k_{eff} A \frac{\Delta T}{d}}}{\frac{\Delta T}{T_{hot}}} = \frac{P_{Max} T_{hot} d}{k_{eff} A \Delta T^2}$$

Using the following identities:

$$P_{Max} = 0.25 \cdot \Delta V_{oc} \cdot I_{sc}$$

$$S_e = \frac{\Delta V_{oc}}{\Delta T}$$

$$\sigma_{eff} = \frac{1}{\rho_{eff}} = \frac{d}{RA} = \frac{\frac{I_{sc}}{\Delta V_{oc}} d}{A}$$

We can rearrange the relative Carnot efficiency formula in a more convenient way:

$$\eta_r = \frac{0.25 \times S^2 \times \sigma_{eff}}{k_{eff}} \times T_h$$

With $k_{eff} \approx 0.7 \frac{W}{mK}$ effective thermal conductivity obtained experimentally, the effective electric conductivity $\sigma_{eff} = 5.1 \frac{A}{V}$ is evaluated from the slope of I-V curve, $S = 3.5 \frac{mV}{K}$ being the Seebeck coefficient in our case and $T_h = 60°C$ the temperature for the hot electrode. This means a Carnot relative efficiency of

$$\eta_r = \frac{0.25 \times (3.5 \times 10^{-3})^2 \times 5.1}{0.7} \times 333.15 \approx 0.75\%$$

Materials and Methods

Hydrogel Synthesis

In a typical synthesis, 4g of polyvinyl alcohol (purchased from Sigma Aldrich Mw 89000-98000, 99+% hydrolyzed) are added to 40ml of Milli-Q pure water and thoroughly stirred. The solution is heated up to 85°C until complete dissolution is achieved, indicated when the solution is completely transparent. Afterwards, the solution is kept stirring for 2h to ensure the homogeneity and the break of polymeric chain. Afterwards, it is left to cool down to room temperature and let at rest and degas overnight. Successively, it is poured in molds and frozen at -20°C for 8h and thawed at room temperature for 16h. This freeze-thaw procedure is repeated for 5 times to ensure complete formation of the polymeric matrix.

Thermoelectrochemical Hydrogel Synthesis

The steps for the preparation of the polymeric solution are repeated as mentioned in the previous section. After the solution reaches room temperature, potassium ferro- and ferri-cyanide redox couples are added in the desired molar concentration. To mitigate ionic interaction between monomers, the salts are first dissolved in a solution of 10%vol Dimethylsulfoxide (DMSO) in Milli-Q pure water to neutralize the charge and then added to the polymeric solution.

Experimental Setup thermo-electrochemical analysis

In a typical experiment, a piece of hydrogel with a 10x10 mm surface area is obtained from a mold. This is placed in a frame, designed to match the hydrogel's dimensions, preventing it from being compressed during experiments and minimizing drying, which could affect the consistency of measurements. Next, two FTO/ITO electrodes are positioned on either side of the hydrogel and secured with screws to ensure good contact at the electrode electrolyte interface. This assembly is then placed onto a Peltier cell used to simulate different heating conditions, with its surface temperature regulated by means of a power supply.

To be noticed that heat is applied to the bottom of the cell, leaving the upper side exposed to the environment without additional cooling, an orientation often referred to as “hot below cold”. [55,56,57]

Thermopower or Seebeck coefficient

To obtain the values of thermopower coefficient, the open circuit voltage and the temperature gradients are monitored in time. The heat is applied to the system and maintained to reach a steady state value. Afterwards, the thermopower is obtained by the ratio of the steady state V_{oc} and the steady temperature difference. To know more about the estimation of thermodiffusive and thermogalvanic contribution refer to section S1. The temperature is monitored by means of two type K thermocouples placed at the interface between the electrode and the electrolyte. The measurements have been repeated multiple times to ensure uniformity of temperature across the hydrogel. During different experiments, the thermocouple placement has been maintained constant thanks to two holes designed in the setup to hold them in position. The open circuit voltage has been measured using a potentiostat (CH Instruments Electrochemical Workstation CHI660D) in two electrode configuration.

Power – Voltage Relation

To obtain the power-voltage relation, a linear sweep voltammetry technique is used. In particular, the heat is applied to the TEC system and maintained for enough time for it to reach a steady state. This condition is achieved when the temperature of the hot and cold electrode is observed to not change significantly in time ($\approx 20min$). After, the voltage is varied in a significant range, meaning from the open-circuit voltage value to zero, to simulate condition where the resistance across the system is modified continuously. The sweep rate is set to be low, i.e. $1mVs^{-1}$, to limit the capacitive effect and give a better estimation of the steady state current. Thus, the current-voltage curve is obtained. This test is run for up to 5 times to ensure that no drift in the measurements is observed, and the final result is an average of all the obtained curves. Finally, the power is simply given by multiplying current and voltage, reaching the final characteristic bell shape reported in Fig. 3a and 3c.

SEM Characterization

The structural characterization of the hydrogel was carried out using scanning electron microscopy (SEM) to assess the morphology and porosity of the as-prepared samples. Hydrogels were first frozen at –20 °C to preserve their internal structure and subsequently freeze-dried for 48 hours to remove residual water without altering the polymer network. The dried samples were then sputter-coated with a thin layer ($\approx 10nm$) of carbon to improve conductivity and minimize charging effects during imaging. SEM analysis was performed using a Zeiss GeminiSEM 300 system under high-vacuum conditions, providing high-resolution images of the hydrogel’s porous architecture.

2D COMSOL simulation

Heat Transfer in the TEC: Heat transfer in the TEC can be expressed as follows:

$$\left(\rho C_p\right)_{eff} \frac{\partial T}{\partial t} + \rho_f \, C_{p,f} \cdot \boldsymbol{u} \, \nabla T - k_{eff} \, \nabla T \; = \; Q$$

$$\left(\rho\, C_p\right)_{eff} = \theta_p\, \rho_p\, C_{p,p} + \left(1-\theta_p\right)\rho_f\, C_{p,f}$$

$$k_{eff} = \theta_p\, k_p + \left(1-\theta_p\right)k_f$$

$$1-\theta_p = \varepsilon_p$$

where, $\varepsilon_p, C_{p,p}, \rho_p$ and k_p are the porosity, heat capacity, density and thermal conductivity of the hydrogel. $C_{p,f}, \rho_f, k_f$ and $\boldsymbol{u}$ are the heat capacity, density, thermal conductivity and velocity of the fluid and the term Q is the heat source. Temperature dependent fluid properties were used.

Fluid Flow in the TEC

The mass conservation of the HyTEC can be expressed as:

$$\frac{d\rho_f}{dt} + \nabla\cdot\ (\rho_f\, \boldsymbol{u}) = 0$$

Momentum conservation was described by the Navier-Stokes-Darcy (Brinkman) equation, expressed as:

$$\frac{1}{\varepsilon_p}\, \rho_f \frac{\partial \boldsymbol{u}}{\partial t} + \rho_f \boldsymbol{u} \frac{\nabla \boldsymbol{u}}{\varepsilon_p^2} = -\nabla p + \nabla K - \frac{\mu}{\kappa}\, \boldsymbol{u} + F$$

where$\left(K = \mu(\nabla \boldsymbol{u}) + (\nabla \mathbf{u})\right)^T$ and the Boussinesq buoyancy term, $F = -\rho_f\ \ g\, \beta_p (T - T_c)$. μ is the kinematic viscosity, κ, the hydraulic permeability of the hydrogel and β_p the thermal expansion coefficient of the fluid. Temperature dependent fluid properties were used.

Mass Transport of Ionic Species

The transport of the diluted ions (oxidized and the reduced species) in the TEC as a result of diffusion due to concentration gradient, migration in the presence of an electric field and convection is given by the Poisson-Nernst-Planck equation:

$$J_i = -D_i\, \nabla c_i - z_i \mu_{mi}\, F\, c_i\, \nabla V + c_i \boldsymbol{u}$$

$$\nabla J_i = R_i$$

where, J_i, R_i, D_i, c_i and z_i are the molar flux vectors, the source term defined by the surface reactions at the electrodes, diffusion coefficient, concentration and charge of species i. The mobility, $\mu_{mi} = \frac{D_i}{RT}$ is given by the Nernst-Einstein equation.

Thermo-Electrochemical Reaction Kinetics

At the electrodes, the thermo-electrochemical reaction is closely related to the overpotential, temperature and concentration of reacting species. The surface reaction rate at the electrode is described using the Butler-Volmer equation.

Anode:

$$J_a = k_0 \exp\left(\frac{E_a}{R}\left(\frac{1}{T_0} - \frac{1}{T_a}\right)\right)\left(C_O^a \exp\left(-\frac{nF(1-\alpha)\eta_a}{RT_a}\right) - C_R^a\, exp\left(\frac{nF\alpha\,\eta_a}{RT_a}\right)\right)$$

Cathode:

$$J_c = k_0 \exp\left(\frac{E_a}{R}\left(\frac{1}{T_0} - \frac{1}{T_c}\right)\right)\left(C_O^c \exp\left(\frac{(nF(1-\alpha)\eta_c)}{RT_c}\right) - C_R^c \exp\left(\frac{nF\alpha\,\eta_c}{RT_c}\right)\right)$$

where $k_0 \exp\left(\frac{E_a}{R}\left(\frac{1}{T_0} - \frac{1}{T}\right)\right)$ is the temperature-dependent rate constant of the oxidation-reduction reaction, n is the number of transferred electrons, α and $1 - \alpha = \beta$ are the electron transfer coefficients, and η_a and η_c are the overpotentials of the anode and cathode. The open circuit voltage of the TEC can be calculated using the following equation:

$$V_{oc} = \eta_c - \eta_a = -\frac{\Delta S_{redox}^0\,(T_a - T_c)}{nF}$$

$$\Delta S_{redox}^0 = S_{[Fe(CN)_6]^{3-}}^0 - S_{[Fe(CN)_6]^{4-}}^0$$

Boundary Conditions

To determine the temperature gradient between the two electrodes we have used two type k thermocouples located at the gel center at the interface with the electrode. For clarity, the anode temperature us labeled as, $T_{anode} = T_h$, and a convective heat flux = $h(T - T_{amb})$ is established at the cathode, where h and T_{amb} are the heat transfer coefficient and the ambient temperature. Apart from the sides representing the two electrodes, other boundaries were set as thermally insulating boundaries:

$$-n \cdot\ q_{wall} = 0$$

To solve the electrochemical kinetics equations, the potential of the anode was set as zero, $\Phi_{anode} = 0$, and the cathode potential was set as the cell voltage, $\Phi_{cathode} = V_{cell}$. Other boundaries were set as insulating boundaries:

$$j_{wall} = 0$$

To solve the mass transport of the chemical species, the flux at the outer walls of the TEC was set to zero:

$$-n \cdot\ (J_i + bf(u)c_i) = 0$$

For the flow equation, the wall of the TEC was set to the no-slip boundary condition $u_{wall} = 0$, and the electrolyte on the top of the TEC was set as the zero-pressure boundary condition $-p_{top} = 0$. The above equations and boundary conditions were solved using COMSOL Multiphysics based on a time-dependent solver, and the data were collected at times when the variation of the current density and concentration of the chemical species were negligible, indicating that the system reached a quasi-steady state. All parameters used in the model are summarized in Table S1. During the calculation, the heat transfer and fluid flow were solved to obtain the distribution of temperature and fluid flow in the TEC. The electric field distribution and the mass transport of the chemical species were solved based on the Poisson-Nernst-Planck equation, which in turn was governed by the heat transfer and fluid flow in the TEC.
